# Applying an Ecohealth Perspective in a State of the Environment Report: Experiences of a Local Public Health Unit in Canada

**DOI:** 10.3390/ijerph120100016

**Published:** 2014-12-23

**Authors:** Steven Lam, Alanna Leffley, Donald C. Cole

**Affiliations:** 1 Department of Population Medicine, University of Guelph, 50 Stone Road East, Guelph, Ontario N1G 2W1, Canada; 2 Grey Bruce Health Unit, 101 17th St E, Owen Sound, Ontario N4K 0A5, Canada; E-Mail: a.leffley@publichealthgreybruce.on.ca; 3 Dalla Lana School of Public Health, University of Toronto, 155 College St, Toronto, Ontario M5T 3M7, Canada; E-Mail: donald.cole@utoronto.ca

**Keywords:** Ecohealth, environmental indicators, public health, state of the environment

## Abstract

We applied an Ecohealth perspective into a State of the Environment report for Grey Bruce Health Unit and summarized environmental and health data relevant for public health practice. We aimed for comprehensiveness in our data compilation, including: standard media categories (e.g., air, water, land); and ecological indicators (e.g., vectors, forests, wetlands). Data sources included both primary (collected by an organization) and secondary (assembled by others). We organized indicators with the Driving forces-Pressure-State-Exposure-Effect-Action (DPSEEA) framework created by the World Health Organization. Indicators of air, water and land quality generally appeared to point towards a healthy state. Vector-borne diseases remained low. Forests and wetlands appeared to be in good condition, however more monitoring data was needed to determine trends in their ecological indicators. Data were not available on biodiversity and fish conditions. The results of our application of the DPSEEA framework suggest that routinely collected environmental and health data can be structured into the framework, though challenges arose due to gaps in data availability, particularly for social and gender analyses. Ecohealth approaches had legitimacy with broader healthy community partners but applying such approaches was a complex undertaking.

## 1. Introduction

Monitoring and addressing environmental health concerns are important aspects of environmental public health practice [1]. Environmental health concerns can be informed through developing environmental health indicators [2] and surveillance systems [3,4]. State of the Environment (SOE) reporting is an internationally accepted approach designed to monitor environmental conditions and changes over time [5,6]. This approach gained popularity in the 1970s, with the Organization for Economic Cooperation and Development (OECD) producing its first SOE report for member countries in 1979 [7], followed by other comprehensive international SOE reports [8,9]. By the early 1980s, a large number of countries had SOE reporting programs. Another example of an international SOE report is the annual State of the World report [10].

The first SOE report in Canada was created at the national level in 1986 [11] followed by others [12,13]. SOE reports in Canada have ranged in scale from local to national, at periodicity ranging from one to 5 years. Campbell and Maclaren (1995) investigated the use of municipal SOE reporting in Canada. Their findings indicate considerable interest in environment reporting [14]. SOE reports were used to diagnose the health of ecosystems, provide early warning signs of dysfunction, identify likely sources of stress, and show areas where environmental management was effective [5,11,14]. A key constituent of a SOE report is indicators, which are simple measures that represent the condition of an environmental issue [14]. SOE reporting became a Ministry of Health and Long-Term Care requirement under the *1997 Mandatory Health Programs and Services Guidelines* (MHPSG) but on January 2009, the *Ontario Public Health Standards and Protocols* replaced the MHPSG so SOE reporting was no longer required. However, some health units and other organizations recognize the importance of SOE reporting and continue to produce SOE reports [15,16].

Interest in understanding the relationship between ecosystems and humans also started in the early 1990’s [17], leading to incorporating more explicit ecosystem approaches to monitoring [18]. Canada has been a leader in applying ecosystem approaches to health and well-being [19]. Ecosystem health, or “Ecohealth”, is a transdisciplinary approach that recognizes the complex biophysical, social, and economic relationships between ecosystems and human health [20]. Ecosystems provide many goods and services that are vital to human health and livelihood. Humans are altering the capacity of healthy ecosystems to deliver goods and services. Knowledge of ecological interdependencies is important for understanding the relationship between the natural environment and human health [21]. While Ecohealth approaches have been incorporated in local SOE reports in Canada [22,23], research on SOE methodologies, incorporation of ecosystem health indicators and their application to municipal SOE reporting appear to be limited, perhaps partly because SOE reporting is not consistently required.

In its first SOE report, the Grey Bruce Health Unit (GBHU) decided to systematically include a wide range of indicators [24]. This paper aims to (1) describe our efforts at adopting an Ecohealth approach to SOE reporting at the local county level (Grey and Bruce counties); (2) present the DPSEEA framework we used for organizing environmental, ecosystem and health data; *and* (3) discuss the challenges encountered and options for future development.

## 2. Methods

### 2.1. Define the Purpose, Target Groups, and Scope

SOE reporting is a dynamic and formative process, and evolves in response to emerging concepts, environmental priorities, and public concerns. It is important that a clear purpose, target groups and scope are defined.

The purposes of Grey-Bruce’s SOE report were to: (1) Provide information on the current state of Grey-Bruce’s natural environment; (2) Describe pressures on the state and driving forces behind those pressures; (3) Describe implications for human health and actions to mitigate adverse health effects; and (4) Increase awareness among decision-makers and the community about the importance of the environment and ecosystems, leading to better natural resource management.

The target groups were the public-at-large and decision-makers at the local level. As this was the first SOE report created by the GBHU, the aim was to create a baseline with available data. The initial scope was limited by the time available (approximately four months), and our desire to assess partner interest in the process and findings.

### 2.2. Geographic Location

**Figure 1:**
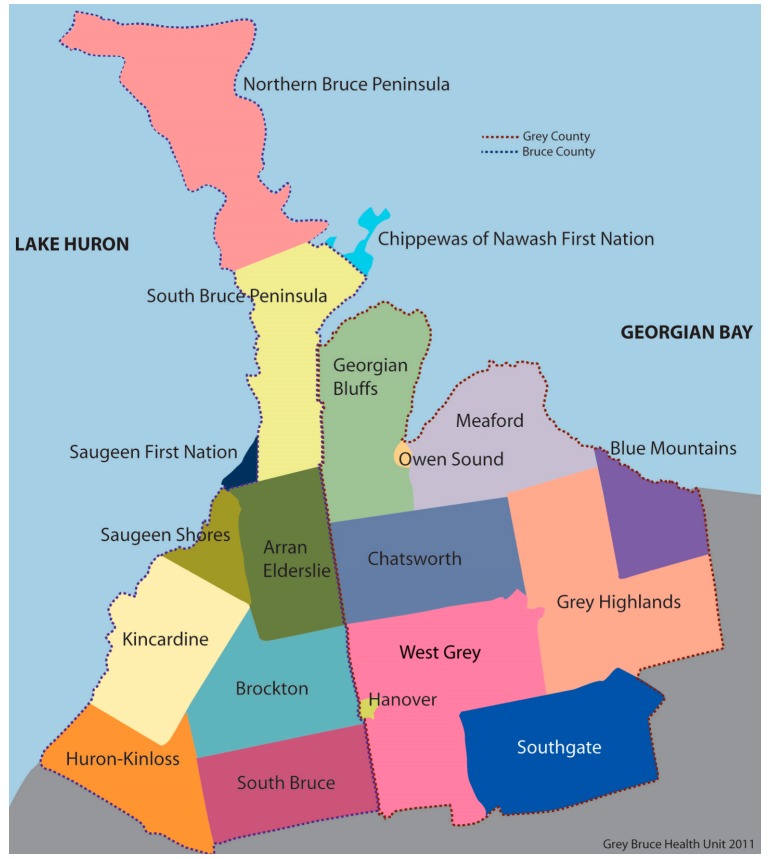
Map of Grey-Bruce municipalities (GBHU 2011).

Grey and Bruce counties are upper-tier municipalities located in Southwestern Ontario, Canada, setting the geographic boundaries for the SOE report (Figure 1) [25]. Together they have a combined population of 158,760 across 8601 km^2^ across 17 municipalities [26]. Located within Grey-Bruce is a United-Nations-designated “world biosphere reserve”, one of only twelve such reserves in Canada. Grey-Bruce has abundant freshwater assets such as Lake Huron, Georgian Bay and many rivers, wetlands and watersheds.

### 2.3. Team

The team included the epidemiologist, environmental health professionals from GBHU, a Master of Public Health student from University of Guelph, and a public health consultant from University of Toronto. Key content expert practitioners from the GBHU, Ministry of the Environment and Climate Change, Ministry of Natural Resources and Forestry, Grey Sauble Conservation Authority, and municipal and county planners provided material or advice for the report, or commented on specific aspects of the report.

### 2.4. Choosing a Framework

A linkage-based framework was selected to incorporate directional relationships relevant to the environment and human health [27]. While a number of conceptual frameworks have been used to guide SOE reporting, the most common frameworks are the Driving force-Pressure-State-Impact-Response (DPSIR), Pressure-State-Response (PSR), or the Driving force-State-Response (DSR), which organize indicators in a casual chain [28,29]. Using the DPSIR framework as an example, driving forces exert pressures on the environment, the state or condition changes as a result of the pressure, impacting health and ecosystems, and hopefully generating responses that address driving forces [30]. A comprehensive SOE report takes into account indicators of stress on environment, indicators of the state of the environment, and indicators of societal response [31]. While the DPSIR, PSR, and DSR frameworks incorporate these elements, they have limitations. They have been criticized for providing a static representation of the environment and ignoring significant interactions between components [31,32,33]. They lack a “bottom line” that would provide the community with an overall assessment of environmental trends [31]. Lastly, these frameworks alone do not provide a sense of immediacy to motivate actions to protect the natural environment, and subsequently health. The immediacy to motivate action can be improved by making “health” a central focus of the SOE report.

**Figure 2:**
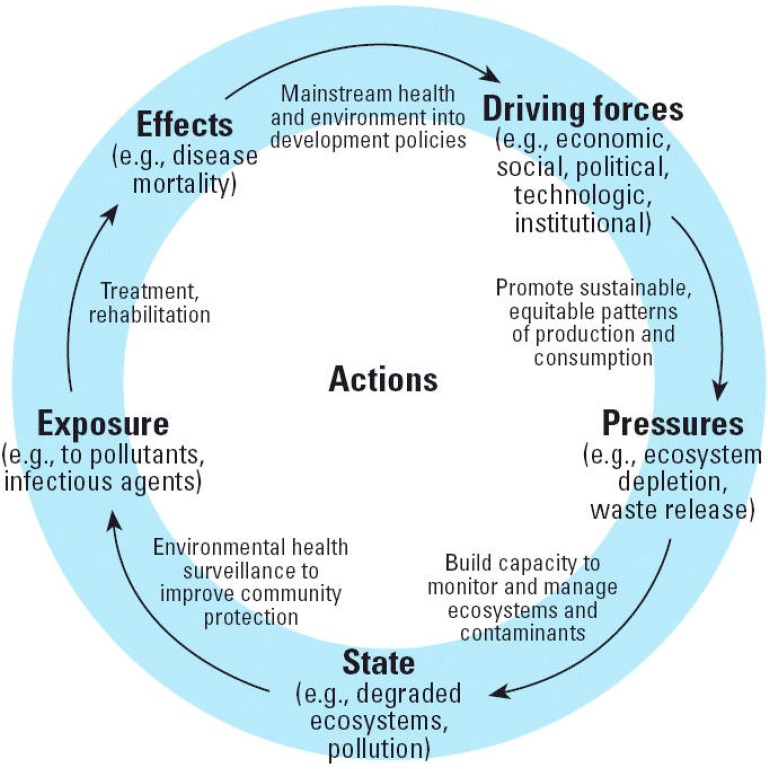
The DPSEEA Framework.

The Driving Force-Pressure-State-Exposure-Effect-Action (DPSEEA) framework is a linkage-based framework created by the World Health Organization to guide the development of environmental indicators (Figure 2) [34]. It aims to describe a comprehensive picture of the way in which various driving forces generate pressures that affect the state of the environment, ultimately affecting health through various exposure pathways [35]. A review of frameworks identified the DPSEEA framework as the most suitable for developing integrated environmental health monitoring [32] and environmental health indicators [33]. The DPSEEA framework can use an ecosystem health approach by showing linkages between environmental exposures and human health outcomes. This approach applies systems thinking by illustrating how broad driving forces impact the health of humans and ecosystems, although it does not fully represent the complex associations between exposures and health [33]. However, given our anticipated scope, the DPSEEA framework was chosen to structure the Grey-Bruce SOE report.

(Source: Carneiro *et al.* 2006 [34], adapted from Corvalan *et al.* 2000 [36]). Reproduced with permission from Environmental Health Perspectives.

### 2.5. Use of the DPSEEA Framework

Next we chose the state component as the initiation point in the framework primarily due to greater available data. To determine exposure and effects, peer-reviewed scientific literature and health status reports were consulted. Actions were determined through an environmental scan of responses by the county organizations which sought to address environmental issues. Driving forces and pressures were determined from peer-reviewed scientific literature, as well as consultations with key experts.

### 2.6. Indicators and Data Collection

Indicators were selected based on data availability and disaggregation to the county level, and whenever possible, indicator validity, responsiveness to change, comparability, and representativeness [37]. Data came from diverse organizations interested in promoting various aspects of environmental health in Grey-Bruce. It included both primary (collected by organization) and secondary (assembled from others) data sources. Examples of primary data sources collected included elements of Grey Bruce Health Unit’s health status reports and adverse water reports database. Examples of secondary data sources included Ministry of the Environment reports and conservation authority reports. Some data collected by the health unit were analyzed specifically for the SOE report, such as private well water bacterial contamination test results.

A specific reference year was chosen (2013) to present current state information. Indicators were grouped into a set or “suite” of indicators representing an aspect relevant to the DPSEEA framework. To represent trends, specific periods were chosen (2003–2013) when data were available. When feasible, trends were analyzed for significance using simple linear regression using Stata 12.1 (Stata Corp., College Station, TX, USA). For example, to determine if the number of adverse water quality results had increased over time, a linear regression was performed setting “adverse water quality results” as the dependent variable and “time” as the independent variable. Statistical significance was determined by interpreting the *p*-value. If significant at *p* < 0.05, the direction of the relationship (+ve or −ve) was reported. We looked at the suite of indicators to assess whether a condition was getting better or worse or staying the same over time.

## 3. Results

### 3.1. Indicators

While indicators were identified for many aspects of the environment, much data required for a comprehensive picture was not available, incomplete, or not representative of the entire region. Given these limitations, a comprehensive state of the environment could not be assembled. Nevertheless, sufficient data were available to indicate that the Grey-Bruce environment was in relatively good condition (see Table 1). To highlight some key examples of each environmental media, for air quality, the concentrations of air pollutants (particulate matter and ozone) consistently met standards and the number of exceedances above provincial standards was low over time. For drinking water quality, the number of adverse water quality incident reports was low over time. For land quality, waste diversion rates have significantly increased over time.

**Figure 3:**
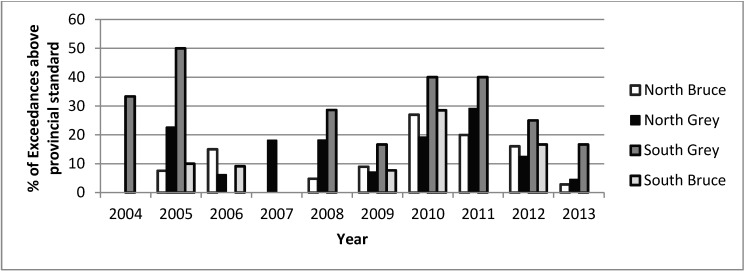
Percentage of beach water sample exceedances above Ontario provincial standard (100 *E. coli*/100 mL) from 2004–2013.

Recreational beach water quality is an indicator that is not generally well reported due to data gaps. The GBHU monitors beach water quality through their beach management program, but attention to any particular beach varies by weather conditions and community concern. The current sampling protocol can be regarded as a valid indicator of near-shore water quality, when sampling is occurring. Refer to Figure 3 for a time trend of percentage of beach water sample exceedances above provincial standard. The number of exceedances appeared to be low, with 2013 being the lowest in 5 years.

Ecological indicators also appeared to be in good condition according to the suite of indicators available (see Table 2). Vector-borne diseases as monitored by the GBHU were of low incidence over time (e.g., Lyme disease, West Nile virus, Eastern Equine Encephalitis Virus). Forests and wetlands are monitored by conservation authorities. The majority of watersheds in the region had “excellent” or “good” forest quality, as well as “excellent” wetland conditions. However more monitoring data are needed to be more comprehensive and to determine trends in the ecological indicators. Although the types of species at risk are known in Grey-Bruce, updated information on the number of species at risk and where they are located was not available and so the status of biodiversity could not be determined. Regarding fish and fish habitat, monitoring data are not yet available at the local level or county level and so the status of fish could not be determined.

**Table 1:** Summary of types of indicators, data sources and key findings for environmental indicators in Grey-Bruce to illustrate how routinely collected data was organized into the state of the environment report. “Over time” indicates the time period from 2003–2013.

Data Collected	Indicator	Summary of Indicator Findings	Data Source(s)
Land			
Energy	Energy consumption and greenhouse gas emission levels	Baseline energy consumption and greenhouse gas emissions were recently established for 2011	Ontario Ministry of Energy *Energy use and greenhouse gas emissions report*
Waste	- Residential waste generated per person - % of waste diverted	- Residential waste generated per person has not changed significantly over time - % of waste diverted has significantly increased over time in Grey County (*p* = 0.009)	Waste Diversion Ontario *Residential Generally Accepted Principles Diversion Rates*
Agriculture	- # of farms and farm area - Farm revenue - # and type of livestock	- # of farms and farm area in Grey-Bruce has decreased from 2006 to 2011 - Farm revenue has increased by nearly 12% from 2006 to 2011 - Grey-Bruce has the most livestock, especially cattle and calves, compared to other counties in Ontario	Statistics Canada *Census of Agriculture*
Soil	- % of soil cover	- % of soil cover appeared * to be increasing over time	Statistics Canada *Census of Agriculture*
Air			
Climate Change	- Rainfall total - Climate average - # of heat alerts - # of emergency department visits due to natural cold or heat	- No significant change in rainfall total over time - Climate averages were steady over the last 20 years - # of heat alerts were low over time, with 1 heat alert reported in 2013 - # of emergency department visits appeared * steady over time	Ministry of the Environment and Climate Change *weather stations* Public Health Ontario *Health profile supplementary data tables*
Air			
Air quality	- Concentrations of particulate matter and ozone - # of particulate matter and ozone exceedances - # of smog days	- Concentrations of particulate matter and ozone consistently met provincial air quality standards ^‡^ - # of exceedances above standards^‡^ was low over time, with 4 exceedances over ozone standards in 2013 and 0 exceedances over particulate matter (2.5 µm) standards in 2013 - # of smog days was low, with zero smog days declared in 2013	-Ministry of the Environment and Climate Change *weather station, smog advisory statistics*
Water			
Drinking water quality	- # of Adverse Water Quality Incident reports for municipal and small drinking water systems - # of Private well water samples submitted for testing - # of adverse results from total coliforms and *E. coli*	- # of Adverse Water Quality Incident reports based on exceedances above Ontario Drinking Water Quality Standards ^‡^ for Grey-Bruce water systems was consistently low over time - # of private well water samples submitted for testing appeared^*^ to be decreasing over time; with adverse results from total coliforms (>5 total coliform) decreasing over time (*p* = 0.004) and adverse results from *E. coli* (>0 *E. coli*) in samples appeared to be steady.	GBHU ^†^ *adverse reports dataset, Laboratory Results Management Application database* Public Health Ontario Laboratories *Water Testing Information System Electronic Notification*
Surface water quality	Benthic invertebrates, total phosphorus and *E. coli* levels	23 out of 34 watersheds (68%) in Grey-Bruce were rated as “excellent” or “good” surface water quality in 2013	Grey Sauble Watershed Report Card 2013 and Saugeen Valley Watershed Report Card 2013
Ground water quality	Nitrite, nitrate and chloride levels	All wells (17) in the Saugeen Valley conservation area were rated as “excellent” ground water quality in 2013	Saugeen Valley Watershed Report Card 2013
Beach water quality	# of exceedances in provincial beach water quality standards	# of exceedances in provincial beach water quality standards ^‡^ appeared to be low from 2004–2013	GBHU ^†^, beach management reports
Water quantity	Water levels of Lake Huron-Michigan	Water levels of Lakes Huron-Michigan appeared * to be decreasing over time	National Oceanic and Atmospheric Administration

* Where “appeared” was used, indicates general visual interpretation with no statistical analysis conducted; ^†^ Abbreviation: GBHU = Grey Bruce Health Unit; ^‡^ Standard or criteria values: Ontario provincial beach water quality standard of 100 *E. coli*/100mL [38], Ontario provincial one-hour ambient air quality criterion for ozone of 80 ppb [39], Canada-wide standard for 24-h averaging time for particulate matter (2.5 µm) of 30 µg/m^3^ [40], Ontario Drinking Water Quality Standards outlines standards for microbiological standards (e.g., zero *E. coli*, less than 5 total coliform) and chemical standards [41].

**Table 2:** Summary of types of indicators, data sources and key findings for ecological indicators in Grey-Bruce to illustrate how routinely collected data was organized into the state of the environment report. “Over time” indicates the time period from 2003–2013.

Data Collected	Indicator	Summary of Ecological Indicator Findings	Data Source(s)
**Vectors**	- # of ticks submitted for testing - # of ticks identified as *Ixodes scapularis* - # of ticks positive for *Borrelia burdorferi* - # of human cases of Lyme disease - # of human cases of WNV ^†^ - # of human cases of EEEV ^†^	- # of ticks submitted for testing increased from 2010 to 2013 - # of ticks identified as *Ixodes scapularis* was low, with 5 ticks identified in 2013 - Zero ticks have tested positive for *Borrelia burgdoferi* from 2010 to 2013 - # of cases of Lyme disease was low from 2005 to 2013, with 2 cases identified in 2013 and identified cases determined to be travel-related - # of cases of WNV ^†^ was low over time, with zero cases identified in 2013 - No human cases of eastern equine encephalitis identified	GBHU ^†^ *vector-borne disease annual reports*
**Biodiversity**	-# of species at risk	-66 species were identified as “species at risk” Grey-Bruce	Ministry of Natural Resources and Forestry *Species at risk*
**Fish**	-Data not available	-Data not available	-Data not available
**Forests**	- % forest cover - % forest interior - % riparian cover	26 out of 34 watersheds (76%) in Grey-Bruce were rated as “excellent” or “good” forest condition in 2013	Grey Sauble Watershed Report Card 2013 and Saugeen Valley Watershed Report Card 2013
**Wetlands**	% wetland cover	7 out of 10 watersheds (70%) in the Saugeen Valley conservation area were rated as “excellent” wetland condition in 2013	Saugeen Valley Watershed Report Card 2013

^†^ Abbreviations: WNV: West Nile virus; EEEV: Eastern Equine Encephalitis Virus; GBHU: Grey Bruce Health Unit.

### 3.2. DPSEEA Framework

Driving forces consistently impact many aspects of the natural environment. They include climate change, population growth and economic growth. Perhaps surprisingly, Grey-Bruce climate indicators showed steady averages over the last 20 years, however measures of variation (e.g., extremes or variance) were not readily available. Population growth in Grey-Bruce was very low at less than 1% from 2006 to 2011. Economic growth generally leads to more resource use which can be accompanied by pollution from agriculture, industries and transportation, unless managed well. There was no measure for tracking economic growth in Grey-Bruce.

Pressures on Grey-Bruce’s environment vary depending on the type of environmental health indicator examined. For water quality indicators and wetland quality indicators, common pressures included agricultural runoff and invasive species. Human health indicators included number of emergency department visits due to natural heat or cold, and number of cases of vector-borne diseases. Many factors play a role in contributing to the healthy environment including local initiatives, community action and provincial policies. In particular, partnerships among different organizations have taken initiatives to tackle environmental issues. To exemplify the DPSEEA framework, we reported selected ecological indicators in Table 3. Vectors, forests and wetlands had adequate data but no suitable exposure or effect indicators were available for forests or wetlands.

**Table 3:** Driving force-Pressure-State-Exposure-Effect-Action framework for selected ecological indicators.

	Vectors	Forests	Wetlands
Driving forces	Climate change, population growth, economic growth		
Pressures	Warming temperatures, agricultural expansion, developmental expansion		
	- Water management - Deforestation	- Invasive species - Demand for resources	- Invasive species - Agricultural runoff - Low water levels - Draining of wetlands
State	# of ticks submitted for testing has increased from 2010 to 2013, # of ticks identified as *Ixodes scapularis* was low, and no ticks have tested positive for *Borrelia burgdoferi*	Forest conditions for the majority of watersheds in Grey-Bruce were rated as “excellent” or “good” condition according to % forest cover, % forest interior, and % riparian cover in 2013	Wetland conditions for the majority of watersheds in the Saugeen Valley conservation area were rated as “excellent condition” according to % wetland cover in 2013
Exposure	Bite of a tick (for Lyme disease) or mosquito (for WNV ^†^ and or EEEV ^†^	N/A	N/A
Effect	- # of cases of Lyme disease was low from 2005 to 2013, with identified cases determined to be travel-related - # of cases of WNV was low over time, with identified cases likely being travel related - No human cases of EEEV identified	N/A	N/A
Action	- Public education campaigns and media releases by GBHU ^†^ - Public health inspector helpdesk - GBHU vector-borne disease monitoring program	- Local initiatives to plant new trees - Conservation authorities forest management plans guided forest management activities, including planting trees - County by-laws to regulate cutting trees within the county	- Conservation authorities participated in educational awareness functions to protect watersheds and wetlands - Ministry of Natural Resources and Forestry encouraged stewardship

^†^ Abbreviations: WNV: West Nile virus; EEEV: Eastern Equine Encephalitis Virus; GBHU: Grey Bruce Health Unit.

## 4. Discussion

In a relatively short time (just under 4 months), the SOE report provided an understanding of Grey-Bruce’s natural environment from a health perspective. Rapport and Singh (2006) argued that making health a central focus can give it an immediacy that previous frameworks lacked. To our knowledge, Grey-Bruce’s SOE report was the first SOE report that used the DPSEEA framework at the county level in Canada. The only other application of the DPSEEA framework to a SOE report was conducted at the country level in New Zealand [42]. We found the DPSEEA framework to be an effective tool for applying an Ecohealth approach as it facilitated understanding of health-environment-development linkages [35]. Although the framework has been criticized for assuming a linear flow from the environmental context to health and ignoring complex linkages between components [33], we found that simple linkages could be easily presented and understood by Board of Health and other practitioner partner audiences.

The DPSEEA framework allowed for the organization of information about environmental conditions, trends and relationships. However, organizing indicators at different steps along the DPSEEA framework was challenging due to lack of data at the local level, especially exposure data (e.g., proportion of population exposed to a contaminant) and health effect data (e.g., morbidity and mortality). Further, although evidence is emerging of the importance of some components such as trees and forests [43], suitable indicators to determine exposure (e.g., cleaner air or visual aesthetics) or health effects (e.g., respiratory or mental health) are not yet developed. Hence the framework can serve as a tool to identify data gaps and areas where more indicator development or monitoring are needed.

The SOE report identified indicators to assess environmental conditions and trends. Further, we used human health indicators (e.g., number of emergency department visits due to natural heat and cold) and sustainability indicators (e.g., waste generation, energy use). The importance of health and sustainability to environmental monitoring has been emphasized in literature [4,37]. While we were guided by criteria for selecting indicators [37], we could not consistently adhere to them because of challenges of scale, data availability and comparability [3,37].

Challenges in widespread implementation of SOE reporting include the lack of common municipal-county indicators and environmental data accessible at the local level [14]. When looking at previous municipal SOE reports in preparation for our report, we found diversity in the indicators used. Each municipality seemed to be developing its own indicators according to data availability. In some cases, data were readily available (e.g., land use, wetland quality, forest quality) and in other cases, data were lacking considerably (e.g., biodiversity, air quality, fish and fish habitat). This means an overall health assessment of the environment could not be provided. Underlying the difficulty in obtaining data is the complex nature of environmental information. The development, testing and eventual reporting of indicators are recommended [37], as well as additional monitoring where data gaps exist. Both could contribute to a more thorough assessment in the future.

Providing information that communicates well to the public and to policy-makers is challenging [4,31]. There needs to be a balance between comprehensiveness (for scientists and environmental planners) and brief summaries (for decision-makers, general public) to maximize the dissemination of information. This framework was intended to be general and broadly applicable to meet the needs of diverse audiences interested in SOE information. Sustainable development decision-making requires the perspectives of all segments of society [6]. When we sought out peer reviews, project design consultations and feedback on the SOE report from relevant practitioner stakeholders, all expressed interest and contributed to our efforts, even though keeping the project manageable in the time frame was challenging.

Considering the complexities of environmental health data, and the different needs of diverse audiences, different products for specific uses [4,6] may be more appropriate for raising awareness of environmental issues. Real-time based products or summary documents with similar functions as the SOE report are options that can meet the needs of broad audiences. However, it is important to integrate reports together to mutually contribute to one another. In the Grey-Bruce SOE report, we included an executive summary as well as provided links to other relevant reports for those interested in more information, but several stakeholders wanted more explicit directions out of the SOE report’s findings.

The SOE reporting process was guided by selected Ecohealth principles including systems thinking, transdisciplinarity and participation. Regarding systems thinking, we aimed to have an integrated human-environment-ecosystem surveillance, and characterize linkages from driving forces to human health effects (as per the DPSEEA framework) in our state of the environment report. We used a transdisciplinary approach through integrating different scientific perspectives from the human health field and environmental health field, primarily those of practitioners and academics. However, we lacked non-academic perspectives, especially input from community members due to the limited scope of the project. This limitation should be addressed in future applications of the DPSEEA framework by engaging community members in the process. We also recognize that other Ecohealth principles, notably gender and social equity were not discussed in the report, primarily because social and gender differences are not highlighted in the DPSEEA framework and available data was not disaggregated. Future SOE reporting could orient towards assessing potential inequities by seeking new data on social and gender differences and their relationships with ecosystems.

Despite interest of public health practitioners in Ecohealth approaches, applying such approaches is a complex undertaking, one for which most health units are not resourced. Ecohealth approaches can gain legitimacy within broader healthy community partnerships, such as the Grey Bruce Healthy Communities process. An Ecohealth approach draws connections between human health and the environment which can open platforms for stakeholders in both the environment and health field to collaborate and commit to a common goal. Community partners can bring different assets, skills and expertise to an SOE project, and can promote the dissemination and uptake of project findings. The experience of the team, combined with the review of primary and secondary data sources resulted in a better understanding of both the potential and limitations of Ecohealth approach to SOE reporting as a public health tool.

## 5. Conclusions

Although we know a great deal about how the natural environment affects us, it is important that we continue to understand what is happening to the environment, why it is happening, what are the consequences and what can be done about it, as organized in the DPSEEA framework. This paper contributes to documenting the monitoring of relationships relevant to the environment, ecosystems and health through reflecting upon our experiences in creating a SOE report [21]. By knowing and sharing more, we can better work to address environmental issues as they arise, contributing to healthier communities. SOE reporting will continue to evolve in response to the changing environmental priorities, public concerns, new findings and new concepts. We encourage other health units and county organizations to try out our approach and report on their experiences.

## References

[1] Eyles J., Furgal C.M. (2002). Indicators in environmental health: Identify and selecting common sets. Can. J. Public Health.

[2] Heo S., Lee J.T. (2013). Study of environmental health problems in Korea using integrated environmental health indicators. Int. J. Environ. Res. Public Health.

[3] Gosselin P., Furgal C.M. (2002). Challenges and directions for environmental public health indicators and surveillance. Can. J. Public Health..

[4] Eyles J. (1999). Health, environmental assessments and population health: Tools for a complex process. Can. J. Public Health.

[5] Jorgensen S.E., Costanza R., Xu F.L. (2005). Handbook of Ecological Indicators for Assessment of Ecosystem Health.

[6] Rump P.C. (1996). State of the Environment Reporting: Source Book of Methods and Approaches.

[7] Sheehy G. (1989). Organizational and Spatial Frameworks for State of the Environment Reporting.

[8] OECD (1985). The State of the Environment.

[9] OECD (1991). The State of the Environment.

[10] The Worldwatch Institute (2014). State of the World 2014: Governing for Sustainability.

[11] Bird P.M., Rapport D.J. (1986). State of the Environment Report for Canada.

[12] Environment Canada (1991). The State of Canada’s Environment.

[13] CCME (1995). State of the Environment Reporting Guidelines for CCME Member Jurisdictions.

[14] Campbell M.E., Maclaren V.W. (1995). An overview of municipal state of the environment reporting in Canada. Can. J. Public Health.

[15] Town of Oakville State of the Environment Report 2012. http://www.oakville.ca/assets/general%20-%20environment/SOER2012.pdf.

[16] City of Windsor City of Windsor’s ROSE: Report on the State of our Environment. http://www.citywindsor.ca/residents/environment/Environmental-Master-Plan/Documents/FINAL%20Rose_2013.pdf.

[17] CPHA (1992). Human and Ecosystem Health. Canadian Perspectives, Canadian Action.

[18] Hancock T. (1993). Health, human development and the community ecosystem: Three ecological models. Health Promot. Int..

[19] Webb J.C., Mergler D., Parks M.W., Saint-Charles J., Spiegel J., Waltner-Toews D., Yassi A., Woollard R.F. (2010). Tools for thoughtful action: The role of ecosystem approaches to health in enhancing public health. Can. J. Public Health.

[20] Blockstein D.E., McManus K.M. (2007). Integrating Environment and Human Health: A report of the Seventh National Conference on Science, Policy and the Environment.

[21] Coutts C., Forkink A., Weiner J. (2014). The portrayal of natural environment in the evolution of the ecological public health paradigm. Int. J. Environ. Res. Public Health.

[22] Harrison G. Renfrew County State of the Environment Report 2004. http://rc-stewardshipcouncil.ca/pdfs/state%20of%20the%20environment.pdf.

[23] York Region Health Services Department (2005). Focus on Our Environment: York Region’s State of the Environment Report.

[24] Lam S., Leffley A., Hart B., Cole D.C. Grey-Bruce’s State of the Environment Report 2014.

[25] Grey Bruce Health Unit About Us. http://www.publichealthgreybruce.on.ca/HOME/Misc/AboutUs.htm.

[26] Statistics Canada Census of Population, Population and Dwelling Counts, for Canada, Provinces and Territories. http://www12.statcan.gc.ca/census-recensement/2011/dp-pd/hlt-fst/pd-pl/Table-Tableau.cfm?LANG=Eng&T=101&S=50&O=A.

[27] Waheed B., Khan F., Veitch B. (2009). Linkage-based frameworks for sustainability assessment: Making a case for Driving Force-Pressure-State-Exposure-Effect-Action (DPSEEA) frameworks. Sustainability.

[28] OECD (1999). Environmental Indicators for Agriculture: Volume 1 Concepts and Frameworks.

[29] EEA (2000). Environmental signals 2000—Environmental assessment report No 6.

[30] Von Schirnding Y. (2002). Health in Sustainable Development Planning: The Role of Indicators.

[31] Rapport D.J., Singh A. (2006). An EcoHealth-based framework for state of the environment reporting. Ecol. Indic..

[32] Liu H.Y., Bartonova A., Pascal M., Smolders R., Skjetne E., Dusinka M. (2002). Approaches to integrated monitoring for environmental health impact assessment. Environ. Health.

[33] Hambling T., Weinstein P., Slaneye D. (2011). A review of frameworks for developing environmental health indicators for climate change and health. Int. J. Environ. Res. Public Health.

[34] Carneiro F.F., Oliveira M.L.C., Netto G.F., Galvao L.A.C., Cancio J.A., Bonini E.M., Corvalan C.F. (2006). Meeting report: Development of environmental health indicators in Brazil and other countries in the Americas. Environ. Health Perspect..

[35] Von Schirnding Y. (2002). Health-environment indicators in the context of sustainable development. Can. J. Public Health.

[36] Corvalán C., Briggs D., Zielhuis G. (2000). Decision-Making in Environmental Health: From Evidence to Action.

[37] Cole D.C., Eyles J., Gibson B.L. (1998). Indicators of human health in ecosystems: What do we measure?. Sci. Total Environ..

[38] Ministry of Environment and Energy (1994). Water Management: Policies, Guidelines, Provincial Water Quality Objectives of the MINISTRY of the ENVIRONMENT.

[39] Ministry of the Environment (2013). Air Quality in Ontario Report for 2011.

[40] CCME (2007). Guidance Document on Achievement Determination: Canada-Wide Standards for Particulate Matter and Ozone.

[41] Ministry of Environment O. Reg. 169/03. http://www.e-laws.gov.on.ca/html/regs/english/elaws_regs_030169_e.htm.

[42] MOH (2009). Environmental Health Indicators for New Zealand 2008.

[43] Trees Ontario (2012). A Healthy Dose of Green: A Prescription for a Healthy Population.

